# Local Gentamicin Fixation with Sprayed Fibrin—An In Vivo Animal Study Reveals New Options to Treat Soft Tissue Infections

**DOI:** 10.3390/jcm12103390

**Published:** 2023-05-10

**Authors:** Meike B. Kejwal, René D. Verboket, Katharina Sommer, Fabian Dust, Dominique Thomas, Philipp Störmann, Johannes Frank, Dirk Henrich, Ingo Marzi, Maren C. Janko

**Affiliations:** 1 Department of Trauma, Hand and Reconstructive Surgery, Hospital of the Goethe University, Goethe University Frankfurt, Theodor-Stern-Kai 7, 60596 Frankfurt am Main, Germany; 2 Institute of Clinical Pharmacology, Goethe University Frankfurt, Theodor-Stern-Kai 7, 60596 Frankfurt am Main, Germany

**Keywords:** acute infections, chronic infections, soft tissue, SSTI, local antibiotics, fibrin-glue-spray technique, animal model

## Abstract

For acute and chronic soft tissue infections, radical surgical debridement is required and is considered the gold standard, along with its immediate systemic antibiotic therapy. Treatment with local antibiotics and/or antibiotic-containing materials is commonly used as an additional tool in clinical practice. Spraying with fibrin and antibiotics is a newer technique that has been studied for some antibiotics. However, for gentamicin, data are not yet available on absorption, optimal application, antibiotic fate at the site and transfer of antibiotic into the blood. In an animal study involving 29 Sprague Dawley rats, 116 back wounds were sprayed with gentamicin using either gentamicin alone or one of two possible spray combinations of gentamicin and fibrin. Simultaneous application of gentamicin and fibrin via a spray system to soft tissue wounds resulted in significant antibiotic concentration over a long period of time. The technique is easy and cost-effective. The systemic crossover was significantly minimized in our study, which may have led to fewer side effects in patients. These results could lead to an improvement in local antibiotic therapy.

## 1. Introduction

The current gold standard treatment for acute and chronic soft tissue infections involves radical surgical wound debridement [1], accompanied by calculated antibiotic therapy. After microbiological diagnostic results are available, targeted antibiotic therapy is initiated [2]. Often, multiple surgical revisions are required to cleanse the wound effectively before secondary wound closure is possible [3]. There are various methods to prevent wound infections, but these procedures do not always lead to success [4,5,6]. In cases of persistent infection, vacuum sealing therapy is one of the procedures that can be used [7,8]. In cases of poor vascularization and critical tissue conditions, local or free microsurgical tissue transfer may be required after clean and well-perfused tissue is obtained from the wound. However, in other cases, after achieving clean and well-perfused tissue, the wound can be closed and complete healing may be achieved [2]. Temporary insertion of antibiotic chains or antibiotic carrier materials to kill germs is one way to improve the infection situation [9,10]. However, all these techniques for wound treatment require a lot of effort, both for the patients with unpleasant reoperations and for the treating physicians. Local antibiotic therapy is not regularly used, although surgical revisions combined with systemic intravenous antibiotic treatments are the most common treatment methods. However, local antibiotic therapy has been shown to result in fewer surgeries, shorter hospital stays, and fewer sepsis-related complications [11,12]. Additionally, local antibiotic therapy increases antibiotic concentrations in the infected area without producing systemic side effects [13]. The most common wound infection of aerobic nature is *Staphylococcus aureus*. Infections with *Streptococcus pyogenes* or *Pseudomonas—aeruginosa* also occur frequently. Other pathogens for typical wound infections are *Escherichia coli, Enterococcus, Proteus and Klebsiella* [14,15]. Gentamicin is mainly effective against Gram-negative bacteria. Good efficacy against *stapylococci, Pseudomonas—aeruginosa* and *E. coli* has been described [16,17]. It was demonstrated by Varga et al. (2014) that gentamycin sponges promote faster wound healing after minor amputations in diabetic patients [9]. The sponges were completely biodegradable, so they did not need to be removed during the second operation. The use of polymethyl methacrylate (Septopal) chains requires successful removal by traction during dressing changes or surgical extractions. These chains are used in osteomyelitis treatment, regardless of the disadvantage of removal, as they provide long-term antibiotic treatment [18,19].

The question of stable retention at the application site, duration of retention and tissue concentration of antibiotics remains in most cases unanswered [20,21]. Many forms of applications have been used, including collagen-gentamicin sponges, chains of polymethyl methacrylate (Septopal), gels containing antibiotics, and pellets containing antibiotics.

In 2016, our group presented a new approach to fixate antibiotics in a wound [22,23,24]. In eight of the nine patients with lower extremity wounds, spraying antibiotics with fibrin after debridement led to good wound healing in combination with systemic antibiotic therapy.

Fibrin spraying is a common method of hemostasizing surfaces, such as the liver and maxillary sinuses [25,26]. However, the field of fixing antibiotics using sprayed fibrin is relatively new. The complex infections of soft tissues or bone-soft tissue combinations, especially in the area of interfaces, are now treated routinely with this procedure. Solid carriers (cement) or collagen carriers (fleece) have the disadvantage that the antibiotic must be released from the solids, which often do not work well over time. However, an immediate effect is evident on all exposed soft tissue surfaces when the antibiotic is sprayed into the wound. In order to avoid antibiotic dilution in the wound, sprayed fibrin glue was applied to fixate the antibiotic in the wound area. Treatment regimes based on this method can be economically and easily applied.

Several clinical cases at our institution have documented the good clinical effects of fibrin-fixed antibiotics [23], but in some cases, more detailed in vivo tests are still pending. For vancomycin and colistin, we were able to show promising results in an animal model, with no tissue-to-blood transition of the antibiotic [22,24]. Such studies are still pending for gentamicin, which is frequently used. 

In order to answer the questions of resorption, the best possible type of application, retention of antibiotic on site and transfer of antibiotic into blood, an animal model was used in this study. We measured the concentration of the antibiotic gentamicin in the soft tissue and blood of the animals to investigate the best type of antibiotic application and its fixation.

## 2. Materials and Methods

### 2.1. Animal Care

All animal experiments were performed in strict compliance with the applicable standards for animal experiments in Germany, and according to the official approval by the Darmstadt Regional Council (project number FK/1133). A total of 29 male rats of the strain Sprague Dawley (Envigo RMS GmbH, Roßdorf, Germany), weighing 335–385 g, were kept in cages with three to four animals each and received food and water ad libitum. For the experiment in 9 groups, 27 animals were required to arrive at *n* = 12 per group. Two animals died during the experiment, so 29 animals were used. Animals that were 8 weeks old were used in the experiment. The cages were housed in constantly ventilated, temperature—(15–21 °C) and light-controlled (14 h day: 10 h night) rooms of the Central Research Institute (ZFE) of the Goethe University Frankfurt am Main (Germany).

### 2.2. Preparation of the Gentamicin/Fibrin Mixture

Gentamicin sulfate 40 mg/mL (Ratiopharm, Ulm, Germany) was dissolved in 9 mL of 0.9% isotonic NaCl solution (Braun, Melsungen, Germany), creating a concentration of 4 mg/mL. A total of 10 mg was applied per wound.

### 2.3. Surgery and Wound Spraying

**Figure 1:**
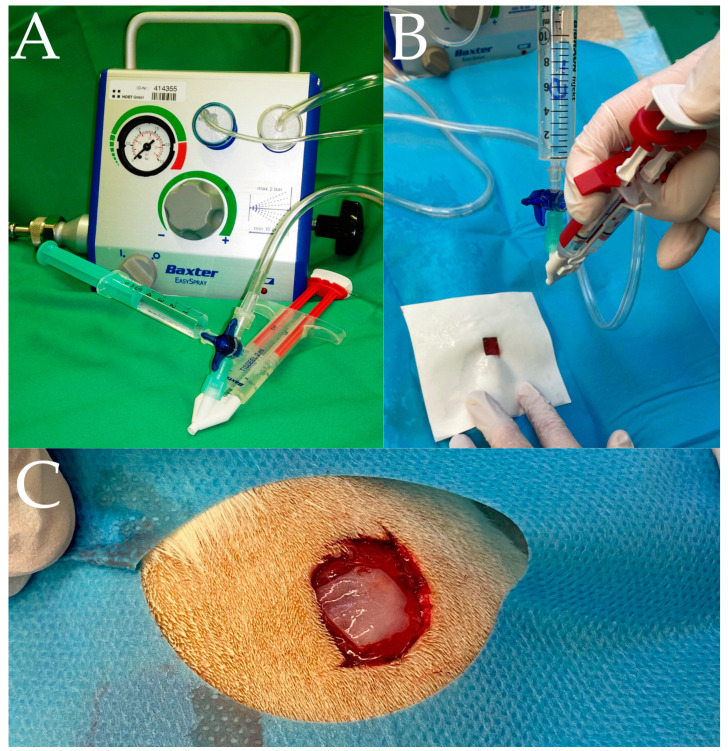
(**A**) Spray setup with TISSUCOL DUO S Immuno© fibrin with DUPLOJECT© syringe attachment and EASYSPRAY© pressure regulator (all Baxter, Heidelberg, Germany). (**B**) Application of gentamicin and fibrin to the defect under coverage of the surrounding tissue. (**C**) Wound 10 × 10 mm, sprayed with gentamicin and fibrin.

Rats were anesthetized by intraperitoneal injection of Ketanest 100 mg/mL (Zoetis, Parsippany, NJ, USA) and Xylavet 20 mg/mL (CP-Pharma, Burgdorf, Germany) at weight-adjusted doses with an injection volume of 1 to 2 mL. The back of the anesthetized rat was shaved and disinfected with Octeniderm (Schülke, Norderstedt, Germany). After sterile covering of the surface with a sterile perforated cloth, four wounds of 100 mm^2^ each were placed under aseptic conditions with a scalpel and scissors to the muscle/fascia level. The fascia was roughened with the scalpel. Then, the wounds were sprayed according to the different groups (Figure 1).

After surgery, animals were placed in single cages and killed depending on the group after 1, 2, and 4 h by an overdose of pentobarbital 500 mg/kg i.p. and the creation of bilateral pneumothoraces. Tissue samples were collected from the wound surfaces. Blood was transferred to EDTA tubes and tissue samples were transferred to 1 mL Eppendorf cups and stored on dry ice at −80 °C in the laboratory until further preparation and measurement.

Gentamicin was sprayed without fixation (G), sequentially with gentamicin and fibrin (GF−), and simultaneously with gentamicin and fibrin (GF+) (Figure 1). Prefilled syringes containing TISSUCOL DUO S Immuno© fibrin (Baxter, Heidelberg, Germany) sealant were used as described in the DUPLOJECT© syringe attachment (Baxter, Heidelberg, Germany) and connected to the EASYSPRAY© pressure regulator (Baxter, Heidelberg, Germany). The distance from the wound to the spray head was 12 cm and the EASYSPRAY© pressure regulator was set to the recommended spray pressure of 1.5–2.0 bar (21.5–28.5 psi). To avoid cross-contamination, the dorsal wounds of a single animal were treated exclusively with one of the three possible application methods. Blood samples were taken to monitor possible systemic uptake. The groups, time points and number of samples used in the experiment are listed in Table 1.

### 2.4. LC-MS/MS-Analysis

For the analysis of gentamicin, 20 µL of an internal standard (tobramycin, 50 µg/mL in water) and 300 µL acetonitrile with 1% formic acid were added to 100 µL of homogenate. After vortexing and centrifugation (5 min, 20,000× *g*), 100 µL of supernatant was used for LC-MS/MS analysis. 

Standards and quality control samples were prepared accordingly with homogenated blank matrix (mice muscle tissue without injected antibiotics) and the corresponding standard (Gen C1 57.6–1612.9 ng; Gen C1a 47.8–1338 ng; Gen C2 67.4–1887 ng; Gen C2a 26.5–742 ng in water).

The LC-MS/MS analysis was carried out using an Agilent 1290 Infinity LC-system with a binary HPLC pump, column oven and autosampler (Agilent, Waldbronn, Germany), coupled with a triple quadrupole mass spectrometer QTRAP 5500 (AB Sciex, Darmstadt, Germany). A Luna^®^ HILIC column (100 × 2 mm, 3 µm, Phenomenex, Aschaffenburg, Germany) was utilized with a flow of 0.35 mL/min and a column temperature of 40 °C. As solvents, 1% formic acid (solvent A) and acetonitrile with 1% formic acid (solvent B) were used with the following gradients (time, percentage B): 0.0 min, 90%; 1.0 min, 90%; 2.0 min, 75%; 2.5 min, 20%; 7.0 min, 20%; 7.3 min, 90%; 11.0 min, 90%. A total of 0.5 µL of each sample was injected.

Data acquisition was performed using Analyst Software 1.7.1, and quantification was performed using MultiQuant Software 3.0.3 (both Sciex, Darmstadt, Germany). Curves were calculated by using linear regression with a weighting factor of 1/x and calibration.

### 2.5. Statistical Analysis

The primary study variable was antibiotic concentration after 1, 2, and 4 h. Each group consisted of 12 samples. For post hoc analysis, a Kruskal–Wallis test with multiple Conover–Iman comparisons was performed to identify the significant features of each group’s results. An effect size of *p* < 0.05 (Bonferroni-Holm corrected) was considered significant. BiAS for Windows version 11.12 dated 01/2021 was used (Epsilon-Verlag, Darmstadt, Germany).

## 3. Results

### 3.1. One Hour in Tissue Time

**Figure 2:**
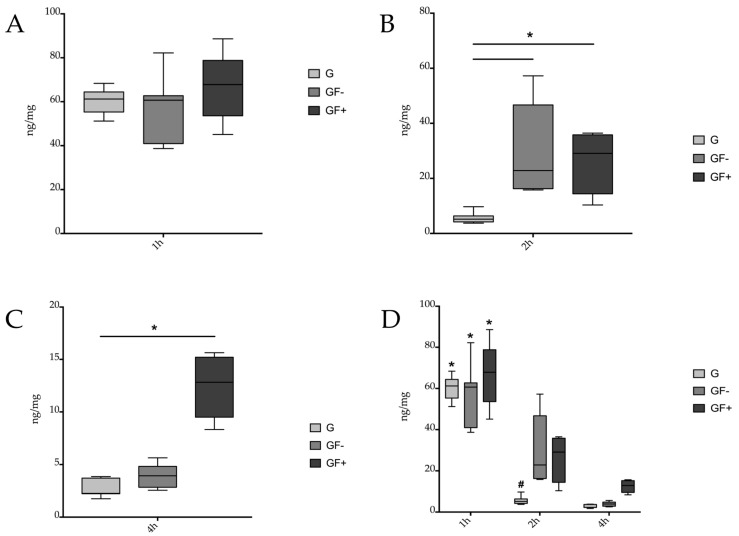
Different spraying techniques of gentamicin over time. Application of gentamicin without fixation (G), consecutive spraying of gentamicin and fibrin (GF−) and simultaneous spraying of gentamicin and fibrin (GF+) after 1 h (**A**). No statistically significant differences were found (*n* = 12). (**B**) After 2 h. Significantly lower values in G group vs. GF− and GF+ (* *p* < 0.05 vs. GF− and GF+). (**C**) After 4 h. Significantly lower values in G group vs. GF+ (* *p* < 0.05 vs. GF+). (**D**) Over the course of the experiment. Significantly higher concentrations after 1 h vs. 4 h in all groups (* *p* < 0.05 vs. G, GF−, GF+ 4 h). After 2 h, values were significantly lower in the G group vs. all groups after 1 h (# *p* < 0.05 vs. G, GF−, GF+ 1 h).

Considering the results of the tissue concentration of gentamicin in ng per mg of tissue in the different groups after one hour, uptake of the antibiotic into the tissue was evident on all sides. Of the 10 mg of gentamicin applied, a mean of 60.688 ng/mg (standard deviation 5.733) was detected after 1 h in the group treated with gentamicin (G) alone. The group with sequentially applied gentamicin and fibrin (GF−) 55.680 ng/mg (standard deviation 15.776) and the group with simultaneously applied gentamicin and fibrin (GF+) 67.629 ng/mg (standard deviation 15.061) showed similar values. No significant differences were found within the 1 h group (Figure 2A). 

### 3.2. Two Hours in Tissue Time

After 2 h, the first differences in the amount of antibiotic in the tissue were observed between the groups. Significantly less gentamicin was detected in the tissues of the group without gentamicin fixation (5.746 ng/mg, standard deviation 1.989, *p* < 0.05 vs. GF− and GF+). The group that received consecutive spraying of gentamicin and fibrin (GF−) (31.867 ng/mg, standard deviation 16.944) and simultaneous spraying of gentamicin and fibrin (GF+) (26.099 ng/mg, standard deviation 10.446) showed significantly higher values (Figure 2B). 

### 3.3. Four Hours in Tissue Time

The greatest differences became apparent after 4 h of tissue time. Here, the group that received simultaneous application of gentamicin and fibrin (GF+) showed the highest values of the antibiotic that still remained in the tissue (12.394 ng/mg, standard deviation 2.785). Lower values were found in the group that received consecutive application of gentamicin and fibrin (GF−) (3.804 ng/mg, standard deviation 1.137), but these were not statistically significantly lower than those in the GF+ group. However, the tissue levels were significantly lower in the group in which only gentamicin was administered (G) (2.816 ng/mg, standard deviation 0.891, *p* < 0.05 vs. GF+) (Figure 2C).

### 3.4. Group Comparison over Time

A comparison of the three groups over the three time points is shown in Figure 2D. Here, a significant decrease in tissue gentamicin concentration was observed between 1 and 4 h in all three groups. (* *p* < 0.05 vs. G, GF−, GF+ 4 h). In the 2 h group, tissue concentration was significantly lower than in the 1 h group only in the gentamicin group without fixation (G) (# *p* < 0.05 vs. G, GF−, GF+ 1 h). The decrease in tissue concentration over time is clearly visible in Figure 2D.

### 3.5. Higher Blood Levels of Gentamicin in G and GF− Groups after 4 h

**Figure 3:**
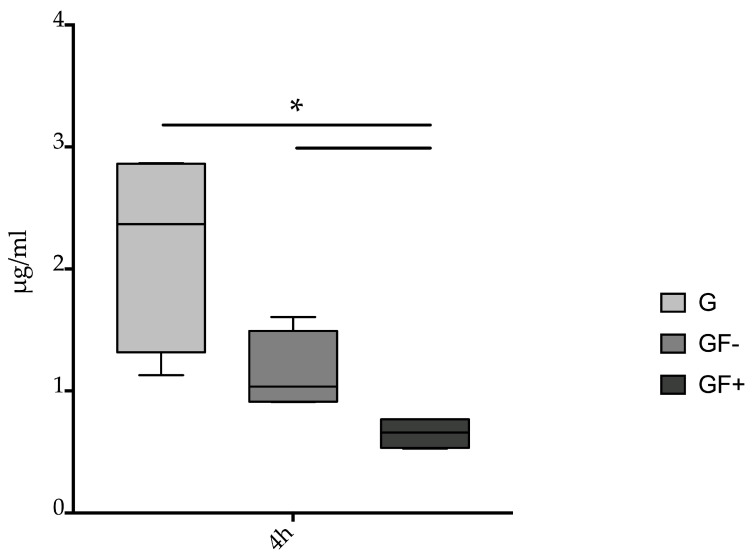
Blood concentration of gentamicin in the animals after 4 h. Application of gentamicin without fixation (G), consecutive spraying of gentamicin and fibrin (GF−) and simultaneous spraying of gentamicin and fibrin (GF+). Significantly lower concentrations of gentamicin in GF+ group (* *p* < 0.05 vs. G, GF−) (*n* = 3).

In the blood samples taken from the animals, the first differences only became apparent after 4 h. At the time points 1 h and 2 h, the measured values of gentamicin or its metabolites were below half the lower limit of quantification (LLOQ 0.598 µg/mL). However, significant differences were observed after 4 h. In the simultaneously sprayed group (GF+) (0.654 µg/mL, standard deviation 0.133), significantly lower levels of gentamicin or its metabolites were found in the blood of the animals than in groups G (2.183 µg/mL, standard deviation 0.839) and GF− (1.147 µg/mL, standard deviation 0.326) (*p* < 0.05) (Figure 3).

## 4. Discussion

With the in vivo experiments shown here, we were able to demonstrate a new technique for antibiotic fixation in wounds for gentamicin, as we did previously for vancomycin. Its application as a spray and fixation with fibrin benefits both the widespread distribution of the antibiotic substance and its ease of application. The antibiotic can be sprayed specifically on a particular area or even on the entire wound site. Even irregular surfaces and soft tissue-bone interfaces are no longer a problem due to the finely misted substances. This concept has already been clinically tested due to its very simple application. In the case of gentamicin, however, the type of application that results in the longest retention of the antibiotic in tissues has never been clearly investigated and established.

We examined the efficacy of spraying gentamicin alone, spraying gentamicin and fibrin sequentially, and simultaneously spraying gentamicin and fibrin to determine which form of application leads to the highest tissue concentration. The data revealed that there are significant differences depending on the mode of application. Spraying with an antibiotic alone or clinical irrigation with an antibiotic solution without any additional fixation had an insufficient effect. Furthermore, it was shown that the binding of *S. aureus* to stabilized fibrin matrices, as used in our trial, promoted a local, macrophage-mediated antimicrobial response [27]. It was demonstrated in this study, as well as in our previous research on vancomycin and colistin [22,24], that the antibiotic levels in the tissue were lowest over the whole observation period if antibiotics were applied solely (Figure 2D). Therefore, the use of antibiotic-containing solutions for wound irrigation is not a viable option and is not clinically accepted [28].

The use of pellets, foams and gels as antibiotic carriers is widespread in clinical practice [28,29]. The release characteristics of antibiotics from these materials show great variation [30]. Antibiotics are often unable to reach the site of infection, especially if administered parenterally. In some cases, the drug concentration in the blood is higher than at the site of infection [31]. Better delivery methods for antibiotic release into tissues need to be developed. An antibiotic containing a bioresorbable gel was found to be significantly more effective in treating infections when Penn-Barwell et al. compared two methods of antibiotic release [13]. There is a large concentration of antibiotic directly on pellets; however, to effectively treat an infection, the antibiotic needs to diffuse into the surrounding tissues. Pellets—such as those made up of gentamicin beads—have an additional major disadvantage because, after the release of the antibiotic, they act as a foreign body and can contribute to persistent infections [13].

Antibiotic spraying allows a large area of infected tissue to be covered, and the antibiotic to be applied in a targeted and uniform manner. A clinically proven nebulizer for spraying fibrin glue is used to spray the antibiotic [32,33]. The compressed air stream in the nebulizer produces a steady stream of nebulized fibrin glue mixed with antibiotic, and a three-way valve can be used to switch between antibiotic and fibrin glue or to nebulize both simultaneously (Figure 1). Thus, it is possible to nebulize only the antibiotic, nebulize the antibiotic and then fix it with nebulized fibrin glue, or nebulize both simultaneously.

With all the application techniques tested in this study, gentamicin sprayed into a wound showed a significant concentration in the wound area after one hour. As a result of the well-perfused tissue in the wound area, the concentration of antibiotics in the wound decreases rapidly (Figure 2B–D). When the antibiotic is fixed with fibrin glue in the tissue, it provides better results in maintaining a high tissue concentration, but again, there are differences between these methods. Gentamicin concentration in the tissue decreases significantly when gentamicin is sprayed sequentially with fibrin, especially after more than two hours (Figure 3). The simultaneous spraying of fibrin glue thus increases the retention time of gentamicin in the tissue, since it prevents the antibiotic from being washed out of the treatment area into the body.

Considering the detection of gentamicin and its metabolic components in the blood of the animals, no metabolites or gentamicin could be systematically detected in the 1 h and 2 h groups. In the 4 h group only, animals receiving gentamicin without fibrin fixation had systemic gentamicin levels twice as high as those receiving gentamicin and fibrin simultaneously.

Therefore, when gentamicin and fibrin are applied simultaneously, the antibiotic substance will remain in the tissue for a longer period and a smaller systemic effect will be produced.

An antibiotic drug fixation technique, such as the one shown here, should not be the only method of treating a local infection. The treatment concept involves comprehensive surgical debridement, nebulizing an antibiotic simultaneously with fibrin glue and next-look surgery, if necessary. It is important to note that intravenous antibiotics are administered as usual [34,35]. 

In addition to treating soft tissue infections, this spray technique can also be used to treat early postoperative soft tissue and bone infections [23,36,37]. The fibrin glue antibiotic mixture is easy to apply, and its procoagulant properties reduce intraoperative bleeding [26]. It is also possible to reduce fluid-filled cavities after the operation due to the adhesive forces of the fibrin. Once purchased, the nebulizer allows for multiple cost-effective uses.

### Study Limitations

In this study, we demonstrated the possibility of antibiotic fibrin spraying in an animal wound model. In an infected wound animal model, however, this procedure has not yet been tested, and comparative studies on other delivery methods are still lacking, but the clinical cases already performed showed good clinical results [23].

## 5. Conclusions

By spraying gentamicin and fibrin onto soft tissue wounds simultaneously, a high level of antibiotic concentration is achieved over a long period of time. A wide range of indications in surgery and wound therapy can be treated with this simple and inexpensive technique. Systemic crossover is minimized, which can lead to fewer side effects in patients. However, further studies are needed, especially for clinical applications in vivo, to demonstrate the therapeutic potential of simultaneously sprayed gentamicin and fibrin.

## Figures and Tables

**Table 1:** Groups, time points and number of samples used in the experiment.

Group	1 h	2 h	4 h
Gentamicin without fixation (G)	*n* = 12	*n* = 12	*n* = 12
Consecutive spraying of gentamicin and fibrin glue (GF−)	*n* = 12	*n* = 12	*n* = 12
Simultaneous spraying of gentamicin and fibrin glue (GF+)	*n* = 12	*n* = 12	*n* = 12

## Data Availability

The data presented in this study are available on request from the corresponding authors.
